# Deciphering the near-complete genome and conducting pan-genome analysis of *Brassica oleracea*

**DOI:** 10.1093/hr/uhaf189

**Published:** 2025-07-16

**Authors:** Qiang Li, Yanhong Fu, Chenhao Zhang, Guoli Zhang, Yuqian Zhao, Ying Wang, Yajing Dou, Lin Gao, Shamsiah Abdullah, Xiao Ma, Yanbin Su, Xiaoming Song

**Affiliations:** Tangshan Key Laboratory of Cruciferous Vegetable Genetics and Breeding, Hebei Key Laboratory of Plant Biotechnology Research and Application, Faculty of Life Science, Tangshan Normal University, Tangshan, Hebei 063000, China; School of Life Sciences/School of Basic Medical Sciences/Library, North China University of Science and Technology, Tangshan, Hebei 063210, China; School of Life Sciences/School of Basic Medical Sciences/Library, North China University of Science and Technology, Tangshan, Hebei 063210, China; Tangshan Key Laboratory of Cruciferous Vegetable Genetics and Breeding, Hebei Key Laboratory of Plant Biotechnology Research and Application, Faculty of Life Science, Tangshan Normal University, Tangshan, Hebei 063000, China; Tangshan Key Laboratory of Cruciferous Vegetable Genetics and Breeding, Hebei Key Laboratory of Plant Biotechnology Research and Application, Faculty of Life Science, Tangshan Normal University, Tangshan, Hebei 063000, China; Tangshan Key Laboratory of Cruciferous Vegetable Genetics and Breeding, Hebei Key Laboratory of Plant Biotechnology Research and Application, Faculty of Life Science, Tangshan Normal University, Tangshan, Hebei 063000, China; Tangshan Key Laboratory of Cruciferous Vegetable Genetics and Breeding, Hebei Key Laboratory of Plant Biotechnology Research and Application, Faculty of Life Science, Tangshan Normal University, Tangshan, Hebei 063000, China; Tangshan Key Laboratory of Cruciferous Vegetable Genetics and Breeding, Hebei Key Laboratory of Plant Biotechnology Research and Application, Faculty of Life Science, Tangshan Normal University, Tangshan, Hebei 063000, China; Faculty of Plantation and Agrotechnology, Universiti Teknologi MARA, Jasin Campus,, Merlimau, Melaka 77300, Malaysia; School of Life Sciences/School of Basic Medical Sciences/Library, North China University of Science and Technology, Tangshan, Hebei 063210, China; Beijing Zhongnong Futong Horticulture Co., LTD, Beijing 100029, China; School of Life Sciences/School of Basic Medical Sciences/Library, North China University of Science and Technology, Tangshan, Hebei 063210, China

**Dear Editor,**

Cabbage, scientifically known as *Brassica oleracea*, is a versatile vegetable from *Brassica* genus of Brassicaceae family. *B. oleracea* is highly valued for its nutritional, ornamental, and medicinal benefits. *B. oleracea* is classified into several morphotypes based on the form of the leaves and growth habit, such as *B. oleracea* var. *capitata* (head cabbage), *B. oleracea* var. *acephala* (kale), *B. oleracea* var. *italica* (broccoli), *B. oleracea* var. *botrytis* (cauliflower), *B. oleracea* var. *gongylodes* (kohlrabi), and *B. oleracea* var. *gemmifera* (Brussels sprouts).

The first genome sequencing of *B. oleracea* released in 2014 was a significant milestone in understanding the genetic makeup of Cabbage [1]. This research provided insights into the dynamics of *Brassica* genome evolution and served as an important resource for *Brassica* vegetable breeding. Recent advances in genomic sequencing have led to the assembly of high-quality genomes for different morphotypes of *B. oleracea* [*1**,*  *2**]*. However, there is still no near-complete genome for *B. oleracea*. Therefore, this study aims to resolve the first high-quality near-complete genome of *B. oleracea*, providing a higher quality genome for comparative and functional genomics research of *B. oleracea*. Moreover, based on this genome, all previously published *B. oleracea* genomes were collected and organized for pan-genome analysis.

To obtain the high-quality *B. oleracea* var. *capitata* genome, we performed the *de novo* genome sequencing using the latest sequencing technologies, including Oxford Nanopore Technology (ONT) ultra-long, PacBio HiFi, Illumina, and Hi-C technology. We selected a type of white cabbage as a representative *B. oleracea* for genomic sequencing research. First, the *B. oleracea* genome was estimated by K-mer using 26.45 Gb data from Illumina sequencing. The estimated genome size was 531 Mb, and the heterozygosity rate was 0.72% (Fig. 1a). The PacBio HiFi sequencer was adopted to generate 30.37 Gb (57.19 X) data with a mean length of 15.05 Kb.

**Figure 1:**
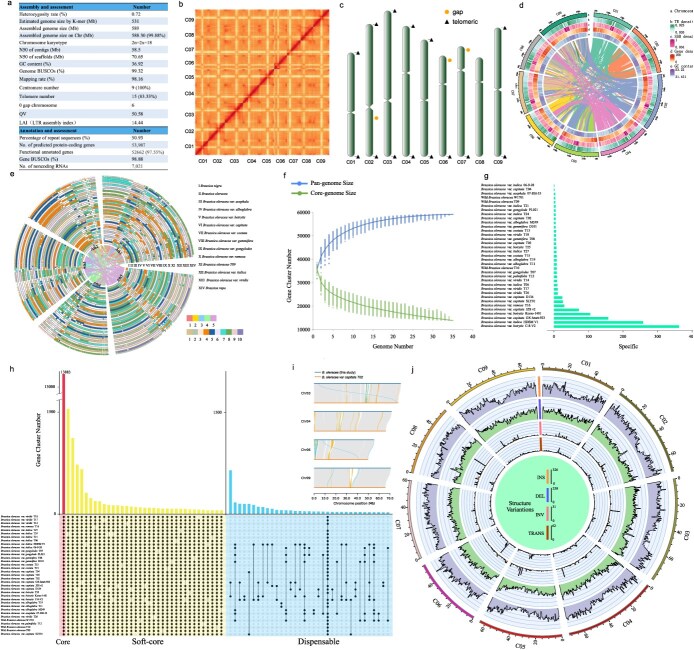
Assessment of *B. oleracea* near-complete genome assembly, annotation, and pan-genome analysis. **(a)** Statistics of *B. oleracea* genome sequencing, assembly and annotation. **(b)** The Hi-C contact map of *B. oleracea* genome assembly. **(c)** Chromosome map of *B. oleracea* genome with telomere, centromere, and gap information. **(d)** The distribution of transposable element, SSR, gene density, and GC content on each chromosome of *B. oleracea*. **(e)** Global alignment of homologous regions. The Ath1–5 regions in the inner circle represent chromosomes 1 to 5 of *A. thaliana* and are defined by five distinct colors. The layers of the outer circle represent the subgenomes of the studied species relative to *A. thaliana*, with the studied species indicated by Roman numerals. The ten colors in the outer circle correspond to the chromosomes of the studied species. **(f)** Variation in the number of gene clusters between the pan-genome and the core genome based on 35 *B. oleracea* species. **(g)** Specific clusters of the pan-genome. **(h)** Core clusters, soft-core clusters and dispensable clusters of the pan-genome. **(i)** The comparative genome visualization map between our genome and *B. oleracea* var *capitata* T02*.*  **(j)** Distribution of structural variations of 34 *B. oleracea* genomes on 9 chromosomes using near-complete genome as reference.

Furthermore, Hi-C technology was used to anchor the assembled sequences to each chromosome, and a total of 54.93 Gb (103.45 X) data were obtained. The assembled genome length was 589.00 Mb, and the 588.30 Mb sequences anchored on the nine chromosomes with anchored ratio was 99.88% (Fig. 1a and b). The high-quality assembled genomes with the contig N50 was 58.50 Mb, and the scaffold N50 reaching 70.65 Mb (Fig. 1a).

Of particular importance is that ONT ultra-long sequences (27.00 Gb, 50.85 X) was used to achieve a near-complete level of genome. Six chromosomes (C01, C03, C04, C05, C08, and C09) have no gap, and the other three chromosomes (C02, C06, and C07) each have only one gap (Fig. 1a and c). This genome consists of nine chromosomes, with a total of 15 telomeres and 9 centromeres were detected. The read-mapping rate exceeds 98.16%, and the coverage is over 99.96% (Fig. 1a). The genome completeness is assessed by BUSCO as 99.32%, and the genome consistency quality value (QV) is 50.58. The genome LTR assembly index value is 14.44, which reached the high-quality reference level (Fig. 1a).

*B. oleracea* var. *capitata* has the most genome assemblies among its morphotypes, driven by its economic importance as a major crop and its status as the first sequenced subspecies in the *B. oleracea* [1]. However, although there are many versions of the genome, none of them have reached near-complete genome level. In this study, the genome of *B. oleracea* var. *capitata* was deciphered at the near-complete genome level. Compared to the genomes previously released, a higher quality and more complete *B. oleracea* genome was obtained. For example, our contig N50 was over 58.5 Mb, which was far larger than the previous report that the best contig is 31.3 Mb. Therefore, this study provides a more accurate and complete genome for the study of gene function and molecular breeding of *B. oleracea*.

Repetitive sequences constituted 50.93% of the *B. oleracea* genome. Specifically, Class I elements (retroelements) accounted for 25.96%, while Class II elements (DNA transposons) represented 24.97% (Fig. 1a and d). A total of 53 987 genes was predicted in *B. oleracea* genome, and 98.88% of BUSCO genes (1614) were detected, indicating high completeness of gene prediction. Among all the predicted genes, over 52 662 (92.16%) genes were annotated by GO, KEGG, KOG, Pfam, SWISS-PROT, TrEMBL, eggNOG, and NR databases. In regard to previous databases, 7021 noncoding RNA were found in *B. oleracea* genome (Fig. 1a).

In addition to our sequenced genome of *B. oleracea* var. *capitata*, we also collected genomes from 11 morphotypes of *B. oleracea*, *B. rapa, B. nigra*, and *Arabidopsis thaliana* to explore the evolutionary history of multiple *B. oleracea* morphotypes and representative Brassicaceae species.

Initially, by observing the homologous gene dot plots between 11 representative *B. oleracea* morphotypes and the reference species *A. thaliana*, we can infer the polyploidization events experienced by the studied species. *A. thaliana* has a relatively clear history of ancient polyploidization events, having undergone not only the whole-genome triplication (WGT) event (γ) that affected most angiosperms but also two additional whole-genome duplication events [1–4]. By examining the homology alignment between the genomes of *B. oleracea* morphotypes and *A. thaliana*, we can observe a clear 3:1 homologous in the gene dot plots between *B. oleracea* morphotypes and *A. thaliana*. This phenomenon also indicates that in addition to sharing ancient polyploidization with *A. thaliana*, the studied *B. oleracea* species have undergone an additional WGT event [1, 5]. Ultimately, a global alignment of the syntenic regions of the studied species is conducted using *A. thaliana* as a reference, and the overall display is presented in the form of a synteny circle map (Fig. 1e).

Based on our *B. oleracea* genome, and other collected 34 genomes from 11 *B. oleracea* morphotypes, we identified 59 086 nonredundant gene clusters, including 1 829 338 genes, which was used for constructing pan genome of *B. oleracea* (Fig. 1f–h). Here, the core cluster was defined as a cluster shared by all 35 genomes. There were 13 883 core clusters containing 723 739 genes (Fig. 1h). Among them, 20 873 genes in our genome are defined as core genes, 17 804 genes have been annotated with Gene Ontology (GO) functions. The function enrichment analysis showed that 12 GO terms were significantly enriched (*p*.adjust value <.05), such as pectin catabolic process, fatty acid biosynthetic process, auxin-activated signaling pathway, and photosynthesis. We further define the clusters present in 34 and 33 genomes as soft-core clusters. Among them, there were 9999 clusters, with a total of 443 960 genes. The 33 997 clusters (57.54%) present in 2–32 genomes contain 655 815 genes (35.85%) and were defined as dispensable clusters. The 1207 clusters existing solely in one genome contain 5824 genes and were defined as specific clusters (Fig. 1g). Among them, 614 genes in our genome are defined as specific genes and 431 genes have been annotated with GO functions. Notably, the GO functional enrichment analysis identified two significantly enriched terms, including nucleic acid binding (GO:0003676) and DNA integration (GO:0015074). However, the inconsistency of different genome versions annotated using different approaches may introduce some technical biases in the classification of gene clusters. Therefore, these specific classifications warrant more rigorous validation through more comprehensive pan-genomic investigations in future studies.

The comparative genome visualization map between our genome and that of *B. oleracea* var. *capitata* T02 reveals distinct improvements in certain regions of our genome. For instance, on chromosome 9, from about 32.5 to 40 Mb, significant enhancements are evident (Fig. 1i)*.* Of course, such regions may also be due to structural variations between the two genomes. In this region, compared to our genome, *B. oleracea* var. *capitata* T02 has undergone 86 deletions, involving 91 genes, of which 87 genes have been annotated with GO functions. Furthermore, the nucleic acid binding and DNA integration terms were also identified by GO functional enrichment analysis. The KEGG annotation for these genes was H+-transporting ATPase (K01535). Furthermore, by aligning 34 *B. oleracea* genomes with our genome as reference, SVs (>50 bp) were classified into four categories, including insertions, deletions, inversions, and translocations. All nonrepetitive SVs include 155 581 insertions, 119 679 deletions, 1022 inversions, and 2289 translocations (Fig. 1j). We found that insertions and deletions are more likely to occur at both ends of chromosomes, while inversions and translocations are randomly distributed on nine chromosomes (Fig. 1j). These structural variations provide a rich data resource for better studying the morphological and genetic diversity of *B. oleracea*.

In conclusion, we present the first high-quality near-complete genome of *B. oleracea* and performed the large-scale pan-genome analysis. This genome and pan-genome analysis provided us with a wealth of data resources for functional genomics studies and molecular breeding of *B. oleracea* or even other Brassicaceae species.

## Data Availability

All genome and annotated data related to this study are available in our TBGR database (http://www.tbgr.org.cn) [3].

## References

[1] Liu S, Liu Y, Yang X. et al. The *Brassica oleracea* genome reveals the asymmetrical evolution of polyploid genomes. Nat Commun. 2014;5:3930 24852848 10.1038/ncomms4930 PMC4279128

[2] Guo N, Wang S, Wang T. et al. A graph-based pan-genome of *Brassica oleracea* provides new insights into its domestication and morphotype diversification. Plant Commun. 2024;5:100791 38168637 10.1016/j.xplc.2023.100791 PMC10873912

[3] Liu Z, Li N, Yu T. et al. The Brassicaceae genome resource (TBGR): a comprehensive genome platform for Brassicaceae plants. Plant Physiol. 2022;190:226–37 35670735 10.1093/plphys/kiac266 PMC9434321

[4] Feng S, Liu Z, Chen H. et al. PHGD: an integrative and user-friendly database for plant hormone-related genes. iMeta. 2024;3:e164 38868516 10.1002/imt2.164 PMC10989150

[5] Wang X, Wang H, Wang J. et al. The genome of the mesopolyploid crop species *Brassica rapa*. Nat Genet. 2011;43:1035–9 21873998 10.1038/ng.919

